# NKCC1 promotes EMT‐like process in GBM via RhoA and Rac1 signaling pathways

**DOI:** 10.1002/jcp.27033

**Published:** 2018-08-29

**Authors:** Haiwen Ma, Tao Li, Zhennan Tao, Long Hai, Luqing Tong, Li Yi, Iruni R. Abeysekera, Peidong Liu, Yang Xie, Jiabo Li, Feng Yuan, Chen Zhang, Yihan Yang, Haolang Ming, Shengping Yu, Xuejun Yang

**Affiliations:** ^1^ Department of Neurosurgery Tianjin Medical University General Hospital Tianjin China; ^2^ Laboratory of Neuro‐Oncology Tianjin Neurological Institute Tianjin China; ^3^ Key Laboratory of Post‐trauma Neuro‐Repair and Regeneration in Central Nervous System, Ministry of Education Tianjin China; ^4^ Tianjin Key Laboratory of Injuries, Variations and Regeneration of Nervous System Tianjin China; ^5^ Department of Physiology and Pathophysiology Tianjin Medical University Tianjin China; ^6^ Department of Neuro‐Oncology The University of Texas MD Anderson Cancer Center Houston Texas; ^7^ Department of Radiation Oncology, Henan Cancer Hospital The Affiliated Cancer Hospital of Zhengzhou University Henan China

**Keywords:** epithelial‐mesenchymal transition (EMT), glioma, invasion and migration, sodium‐potassium‐chloride cotransporter 1 (NKCC1), Rac1 and RhoA

## Abstract

Glioblastoma is the most common and lethal primary intracranial tumor. As the key regulator of tumor cell volume, sodium‐potassium‐chloride cotransporter 1 (NKCC1) expression increases along with the malignancy of the glioma, and NKCC1 has been implicated in glioblastoma invasion. However, little is known about the role of NKCC1 in the epithelial‐mesenchymal transition‐like process in gliomas. We noticed that aberrantly elevated expression of NKCC1 leads to changes in the shape, polarity, and adhesion of cells in glioma. Here, we investigated whether NKCC1 promotes an epithelial–mesenchymal transition (EMT)‐like process in gliomas via the RhoA and Rac1 signaling pathways. Pharmacological inhibition and knockdown of NKCC1 both decrease the expressions of mesenchymal markers, such as N‐cadherin, vimentin, and snail, whereas these treatments increase the expression of the epithelial marker E‐cadherin. These findings indicate that NKCC1 promotes an EMT‐like process in gliomas. The underlying mechanism is the facilitation of the binding of Rac1 and RhoA to GTP by NKCC1, which results in a significant enhancement of the EMT‐like process. Specific inhibition or knockdown of NKCC1 both attenuate activated Rac1 and RhoA, and the pharmacological inhibitions of Rac1 and RhoA both impair the invasion and migration abilities of gliomas. Furthermore, we illustrated that NKCC1 knockdown abolished the dissemination and spread of glioma cells in a nude mouse intracranial model. These findings suggest that elevated NKCC1 activity acts in the regulation of an EMT‐like process in gliomas, and thus provides a novel therapeutic strategy for targeting the invasiveness of gliomas, which might help to inhibit the spread of malignant intracranial tumors.

**Abbreviations**
CCK‐8: cell counting kit‐8
CTCs: circulating tumor cells
DMSO: dimethyl sulfoxide
EGF: epidermal growth factor
EMT‐like: epithelial‐to‐mesenchymal (‐like) transition
GAPDH: glyceraldehyde 3‐phosphate dehydrogenase
GBM: glioblastoma multiforme
MMP2: matrix metallopeptidase 2
NKCC1: sodium‐potassium‐chloride cotransporter 1
PDX: patient‐derived xenograft
SLC12A2: solute carrier family 12 member 2
TCGA: The Cancer Genome Atlas
TMA: tissue microarray
WHO: World Health Organization

## 1 INTRODUCTION

Glioblastoma multiforme (GBM) is one of the most lethal human tumors. Most GBM patients without treatment die within 3 months of diagnosis (Stupp et al., 2005, 2009). Even with surgical intervention combined with chemo‐ and radiation therapies, the median survival time for GBM is still 12–16 months (Preusser et al., 2011). Due to its highly invasive nature, GBM is the most aggressive form of brain cancer, which impedes the surgical removal of all tumor cells and makes relapse inevitable. Hence, to improve the current poor prognosis of GBM patients, the identification of effective therapeutic targets to impede intracranial invasion and the spread of tumor cells is imperative.

The sodium‐potassium‐chloride cotransporter 1 (NKCC1), also known as solute carrier family 12 member 2 (SLC12A2), transports one sodium ion, one potassium ion, and two chloride ions across the plasma membrane and is sensitive to loop diuretics. It has been reported that NKCC1 is highly expressed in gliomas and is tightly related to many malignancies. Pharmacological inhibition of ion cotransport reduces the ability of tumors to spread, and the agonists of the ion cotransport markedly increase their spread (Cong, Zhu, Kuo, Hu, & Sun, 2015). Moreover, the inhibition of NKCC1 accelerates temozolomide‐mediated apoptosis in glioblastoma cells (Algharabil et al., 2012). The overexpression of NKCC1 induces cell proliferation and phenotypic transformation in mouse fibroblasts (Panet, Marcus, & Atlan, 2000). Moreover, it has been demonstrated that NKCC1 plays an important role in the proliferation of gastric and prostate cancer cells (Hiraoka et al., 2010; Shiozaki et al., 2006). Shiozaki et al. (2014) also suggested that NKCC1 plays an important role in cell cycle progression in human esophageal squamous cell carcinomas. It is well known that the infiltrative growth pattern is one of the most significant characteristics of malignant gliomas, and the transformation ability of tumor cells to cross the narrow extracellular space is crucial for infiltration (Cuddapah & Sontheimer, 2011). This evidence indicates that NKCC1 is a key protein in carcinogenesis and that NKCC1 could become a new therapeutic target for the malignancy, but the related potential mechanisms of carcinogenesis have not yet been elucidated.

Epithelial–mesenchymal transition (EMT) is a complex process involving a high level of phenotypic plasticity that enables epithelial cells to lose their cell polarity and cell adhesion and gain migratory and invasive properties, which leads to a mesenchymal phenotype. Moreover, EMT is thought to be essential for tumor metastasis in systemic cancers (Tam & Weinberg, 2013). Although some debates still exist regarding the EMT concept in gliomas, an “EMT‐like” process is accepted in brain tumors (Iser, Pereira, Lenz, & Wink, 2017). The EMT‐like process refers to an intermediate phenotype that turns into a phenotype that is less epithelial and more mesenchymal (Iser et al., 2017). Although glioblastoma patients rarely exhibit extracranial metastasis complications, it is surprising that approximately 20% of GBM patients have detectable levels of circulating tumor cells in their blood (Awan et al., 2015). Hence, the EMT‐like process has been proposed to play a very important role in the spread and metastasis of GBM. Several pathways have been found to regulate the EMT, including NF‐κB, Wnt, PI3k/Akt, and so forth (Maier, Traenkle, & Rothbauer, 2016). Recently, additional studies have demonstrated that the Rac1 and RhoA pathways play pivotal roles in the EMT process (Gulhati et al., 2011; Schiapparelli et al., 2017; Zhang et al., 2014).

In the initial steps of metastasis, some important regulatory factors involving the activation of small GTPases, including RhoA and Rac1, play a crucial role in actin cytoskeletal rearrangement and cell migration (Hall, 1998). For example, Rho GTPases regulate many important cellular processes including cytoskeletal remodeling (Ellenbroek & Collard, 2007). Bar‐Sagi and Hall (2000) support the notion that crosstalk between Ras and Rho proteins is involved in several biological processes, including cell transformation, cell migration, and the EMT. In summary, Rho GTPases play an important role in the EMT process in tumors, and this process is closely correlated with the malignancy of tumors. In colorectal carcinoma, Rac1 and RhoA act as an intercurrent signaling pathway that regulates the EMT and tumor metastasis (Gulhati et al., 2011). Additionally, in breast cancer, Rac1, RhoA, and cdc42 also serve as regulators of the EMT (Zhang et al., 2014).

In this study, we suggest that NKCC1 was highly expressed in GBMs with surrounding multifocal infiltration and spread, and NKCC1 promoted the invasion and migration of gliomas. Furthermore, NKCC1 promoted the EMT‐like process in gliomas via the RhoA and Rac1 signaling pathways; inhibition of NKCC1 significantly impaired the aggressive progression of gliomas in vitro and in vivo, which indicated that NKCC1 serves as a potential therapeutic target for the inhibition of the dissemination of gliomas.

## 2 MATERIALS AND METHODS

### 2.1 Cell culture and reagents

Two human malignant glioma cell lines (U87‐MG, SNB19) were purchased from the Institute of Biochemistry and Cell Biology (Shanghai, China) and were cultured in Dulbecco modified Eagle medium (Gibco, Invitrogen Inc., Carlsbad, CA) supplemented with 10% fetal bovine serum (Gibco) and incubated at 37℃ in 5% CO_2_. NKCC1 rabbit mAb (No.ab59791 WB 1:1,000, IHC 1:100), NKCC1 goat mAb (No.ab99558 IF 1:100), vinculin mouse mAb (No.ab129002 IF 1:100), MMP‐2 rabbit mAb (No.ab37150 WB 1:1000) were purchased from Abcam (UK), E‐cadherin ribbit mAb (No.3195P WB 1:1,000), N‐cadherin rabbit mAb (No.13116S WB 1:1,000 IF 1:100), snail rabbit mAb (No.3879P WB 1:1,000 IF 1:100), vimentin rabbit mAb (No.5741P WB 1:1,000 IF 1:100) were obtained from Cell Signaling Technology (Danvers, MA). B‐actin mouse mAb (15V70207 WB 1:200‐1,000), GADPH mouse mAb (TA309157 WB 1:2,000) was obtained from ZSGB‐BIO (China).

NKCC1 inhibitor bumetanide (BMT), Rac1 inhibitor NSC25766, RhoA inhibitor Y27632 from Selleck (China). Epidermal growth factor (EGF) cytokines for cell activated were purchased from Gibco.

### 2.2 Sample collection

Five hundred and forty samples of GBM from The Cancer Genome Atlas (TCGA, https://tcgadata.nci.nih.gov). Clinical tissue chip and image data were obtained from the Department of Neurosurgery at Tianjin Medical University General Hospital (Supporting Information Table S1). All the samples were histologically graded according to the 2007 World Health Organization (WHO) Classification of Nervous System Tumors. A written informed consent was obtained from all donors and their relatives. The study was carried out in accordance with the principles of the Helsinki Declaration and approved by the ethical committee at Tianjin Medical University General Hospital.

### 2.3 Western blot analysis

Total cell lysate was prepared as described previously [13]. After denaturation, the proteins were separated by gel electrophoresis using 10% or 12% sodium dodecyl sulfate‐polyacrylamide gel electrophoresis and electrotransferred to polyvinylidene fluoride membranes (Millipore, Billerica, MA). After being washed three times by Tris‐buffered Saline with Tween 20 (TBST), the membrane was blocked by 5% bovine serum albumin (BSA) for 1 hr at 37°C. Then, first antibodies were incubated overnight at 4°C. The membrane was again washed three times with TBST before incubation with the secondary antibody (goat anti‐rabbit/mouse IgG 1:2,000) for 1 hr at room temperature and was washed a third time with TBST. The proteins were detected by the G:BOX (Syngene Company, UK) using Chemiluminescent HRP Substrate (Millipore).

### 2.4 Tumor cell proliferation assay (cell counting kit‐8 assay)

U87‐MG, SNB19 cells (4 × 10^3^ cells per well) were seeded into 96‐well plates. After 24 hr or 72 hr treatment by BMT, a specific inhibitor of NKCC1, 10 μl of CCK‐8 (Dojindo Laboratories, Kumamoto, Japan) was added to each well and incubated for 2 hr at 37°C. The absorbance at 450 nm was measured on a Synergy 2 microplate reader (BioTek).

### 2.5 Immunofluorescence analysis

Different cells were seeded onto cover slips in a 12‐well plate overnight. The cells were washed with phosphate buffer saline (PBS) three times and fixed with 4% paraformaldehyde for 10 min. Then, they were permeabilized with 0.1% Triton X‐100 for 10 min (except membrane antigen) and blocked in 5% BSA at room temperature for 30 min. They were then incubated with a primary antibody at 4°C overnight. After being rewarmed for 1 hr, the samples were washed with PBS three times and incubated with specific secondary antibodies for 1 hr at 37°C. After washing three times with PBS, the nuclei were stained with 4',6‐Diamidino‐2‐Phenylindole (DAPI) for 5 min at room temperature. Immunofluorescence was observed using fluorescence microscopy (Olympus, Japan).

### 2.6 Transwell and wound‐healing assay

Cell migration and invasion assays were carried out as described previously (Zhu et al., 2014).

### 2.7 Pull‐down assays

RhoA and Rac1 activity was assessed using the GST‐tagged Rho‐binding domain of Rhotekin (TRBD) and the GST‐tagged p21 binding domain of PAK1 (GST‐PBD) pull‐down assays, respectively, as described previously (Gulhati et al., 2011; O’Connor, Nguyen, & Mercurio, 2000). Briefly, the cells were grown to ~70% confluency in regular growth medium and stimulated with EGF (50 ng/ml) for 5 min. For BMT treatment, the cells were preincubated in 50 μM BMT for 24 hr. The RhoA monoclonal antibody was from Cell Signaling Technology and the Rac1 monoclonal antibody was from Millipore.

### 2.8 Gelatin zymography

Equal numbers of cells were seeded in serum free conditions. Supernatants were collected, normalized for total protein concentration, mixed with sample buffer (Invitrogen), and analyzed by electrophoresis with a 10% zymogram gel (Invitrogen) for 90 min. The gel was developed according to the manufacturer's instructions and stained with Coomassie Blue (Invitrogen).

### 2.9 Lentiviral transfection

Lentiviral shRNA constructs were obtained from Genechem Co., Ltd., China. ShRNA sequences were as follows: TCAGGCTCTATGTAAGGAC (shNKCC1‐1), CACTATCGTAACAGAGCTA (shNKCC1‐2). Firefly Luciferase lentiviral particles were obtained from Genechem Co., Ltd. Cells were transfected with either lentiviral particles, following the manufacturer's recommendations. After infection, stable cell clones expressing the shRNA constructs were isolated by selection with 5 μg/ml puromycin solution. The cells were collected for further experiments at 48 hr after the transfection.

### 2.10 Immunohistochemical staining

Paraffin‐embedded tumors were sectioned and dewaxed. After antigen retrieval (treated in 10 mmol/L citrate buffer for 20 min at 95°C), sections were cleared of endogenous peroxidase activity by incubation with 3% H_2_O_2_ for 15 min and blocked with 5% BSA for 30 min at 37°C. They were then incubated with primary antibodies at 4°C overnight. The next day, after rewarming for 1 hr, sections were incubated with biotinylated secondary antibodies for 1 hr at 37°C. They were then incubated with ABC‐peroxidase for 1 hr before sections were colored using a DAB Kit (ZSGB‐BIO) and counterstained with haematoxylin. After dehydration, sections were examined using a light microscope.

### 2.11 Animals and intracranial xenograft model

Animal experiments were approved by the Ethical Committee in Tianjin Medical University General Hospital. A total of eight female immunocompromised nude mice, aged 4 weeks, were randomly divided into two groups (four mice each group) for intracranial implantation of shNKCC1 (concentration of shRNA =  10^8^ TU/ml) and scramble U87‐MG cells with luciferase expression, respectively. The mice were anaesthetized, placed in a stereotactic frame (RWD Life Science, China), and injected with specific numbers of glioma cells in 10 μl of PBS through a 27‐gauge needle at 2 mm lateral and posterior to the bregma and 3 mm below the dura. Cell suspension was injected slowly in 20 min. Then the needle was kept in the injection site for 5 min before removing it. The mice were housed under pathogen‐free conditions in a barrier animal facility. Tumor cells bioluminescence imaging was performed to assess xenograft formation at 7/14/21 days after implantation by using the IVIS Spectrum Live Imaging System (Tianjin Medical University, China). Image calibration and visualization were performed using Live Image 4.4 Software.

### 2.12 Statistical analysis

All quantified data represent an average of at least triplicate experiments, unless otherwise indicated. Standard deviations were calculated. All statistical analyses were performed using GraphPad Prism 6.0 (GraphPad Software, La Jolla, CA). Comparisons among groups were performed using unpaired Student *t* tests. *p* < 0.05 was considered to be statistically significant.

## 3 RESULTS

### 3.1 Overexpressed NKCC1 drove multifocal tumor infiltration and spread and was correlated with shorter survival in GBM

**Figure 1:**
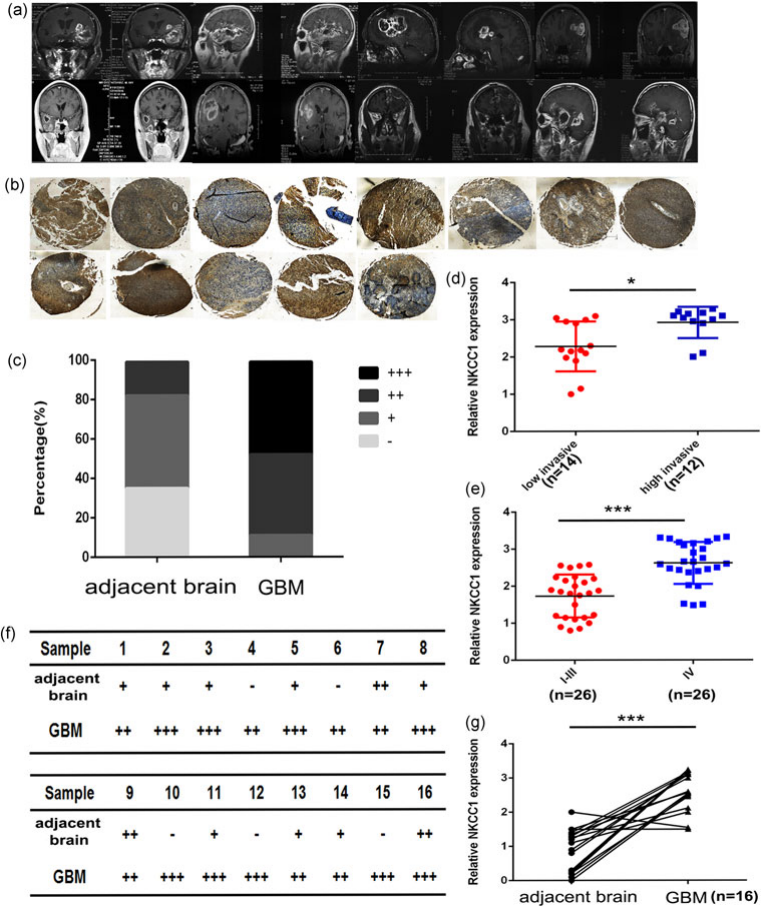
NKCC1 expression was increased in GBM patients who exhibited intracranial multifocal infiltrative features. (a) MRI images of eight GBM patients who exhibited surrounding multifocal infiltration, which was characterized by the invasion of adjacent structures and the spread to other parts of the brain to form multiple regional small satellite lesions. (b) NKCC1 expression was analyzed in the cores and borders in highly invasive GBM patients by tissue microarray. c: core, b: border. (d) NKCC1 expression in the highly invasive GBM group and the lowly invasive GBM group was detected by immunohistochemistry analyses (*p* = 0.0129; *n* = 26). (c,f,g) IHC analyses of NKCC1 expression in 16 paired clinical GBM/adjacent brain tissues. Quantification of NKCC1 expression in the IHC analysis performed in f. The intensity of immunostaining was graded as follows: −: negative; +: weakly positive (light brown); ++: moderately positive (brown); and +++: strongly positive (dark brown). Three pathologists who were blinded to the clinical data independently scored all slides. (e) TMA data showing the NKCC1 expression in GBMs and lower grade gliomas (*n* = 52). GBM: glioblastoma multiforme; MRI: magnetic resonance imaging; NKCC1: sodium‐potassium‐chloride cotransporter 1; TMA: tissue microarray [Color figure can be viewed at wileyonlinelibrary.com]

Based on clinical investigation, the tumor tissues of GBM patients (*n* = 12) that exhibited severe intracranial spread and multifocal features strongly expressed NKCC1. Moreover, tissue microarray (TMA) data revealed that NKCC1 was highly expressed in the cores and borders of tumors (Figure 1b) in eight GBM patients with surrounding multifocal infiltration that was characterized by the invasion of adjacent structures and spread to other parts of the brain to form multiple regional small satellite lesions (Figure 1a). Nevertheless, other GBM patients had relatively circumscribed margins that often gave the impression of demarcation on gross inspection and exhibited lower NKCC1 expression (*p* = 0.0129; Figure 1d). Additionally, a TMA (*n* = 52; WHO I‐III lower grade glioma, 26; WHO IV higher grade glioma, 26; see Supporting Information Figure S1A) revealed that NKCC1 is expressed in glioma tissues at a higher level in higher grade compared with lower grade gliomas (Figure 1e). From another point of view, the expression of NKCC1 of GBM was higher than adjacent brain tissue (Figure 1c,f,g). Immunohistochemistry (ICH) samples from high grade glioma (HGG) groups taken from the Human Protein Atlas stained stronger for NKCC1 than did samples from LGG groups (Supporting Information Figure S1B). Together, these findings strongly indicate that NKCC1 plays a critical role in intracranial GBM dissemination and is related to patient prognosis.

### 3.2 NKCC1 was significantly correlated with a mesenchymal phenotype in GBM

**Figure 2:**
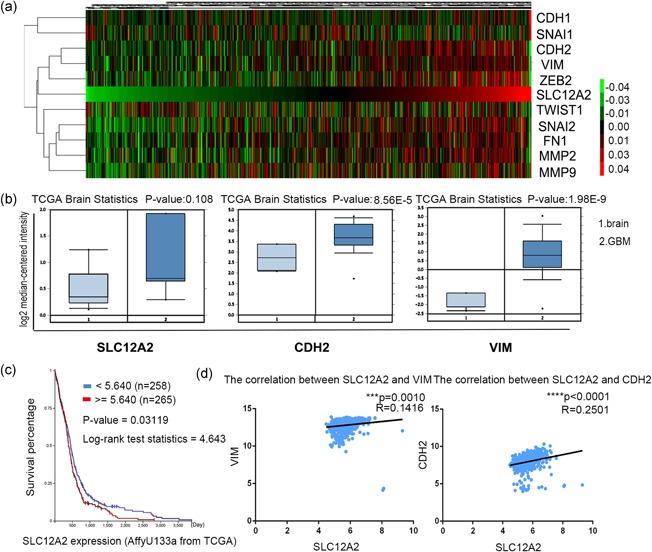
NKCC1 expression was increased in GBM, and it was positively correlated with mesenchymal markers in GBM (a) Unsupervised hierarchical clustering analysis from the TCGA datasets was performed to show that the distinctive features of EMT‐related gene expression were changed in parallel with SLC12A2 expression in GBM tissues. (b) NKCC1/CDH2/VIM expression was analyzed in GBM tissues and non‐tumor brain tissues from the TCGA data set. (c) Kaplan‐Meier survival curve analysis indicated that GBM patients with NKCC1 overexpression had a significantly shorter overall survival (*p* = 0.03119). (d) Pearson correlation analysis of the relationship between SLC12A2 and CDH2/VIM mRNA expression in the TCGA data sets. EMT: epithelial–mesenchymal transition; GBM: glioblastoma multiforme; NKCC1: sodium‐potassium‐chloride cotransporter 1; TCGA: The Cancer Genome Atlas [Color figure can be viewed at wileyonlinelibrary.com]

Based on the TCGA database, the NKCC1 expression was higher in GBM tissues than in normal brain tissues (Figure 2b). Next, we evaluated the prognostic values of NKCC1 in GBM with Kaplan‐Meier survival curve analysis. The patients with higher NKCC1 expression levels had shorter overall survival (*p*‐value = 0.03119; Figure 2c). Then, we examined the expression of EMT‐related genes in GBM by analyzing the TCGA data set. The expressions of the mesenchymal marker vimentin and N‐cadherin were significantly increased in GBM (Figure 2c). Unsupervised hierarchical clustering was performed to analyze the expressions of epithelial and mesenchymal genes. The results revealed that GBM was associated with greater expression of almost all of the mesenchymal genes and lower expression of epithelial genes, and this distinction changed in parallel with SLC12A2 expression (Figure 2a). Furthermore, a correlation analysis of the TCGA data indicated that NKCC1 expression was positively correlated with vimentin and N‐cadherin (Figure 2d). These data suggested that NKCC1 is involved in the EMT‐like process in gliomas.

### 3.3 Inhibition of NKCC1 decreased invasion and migration

**Figure 3:**
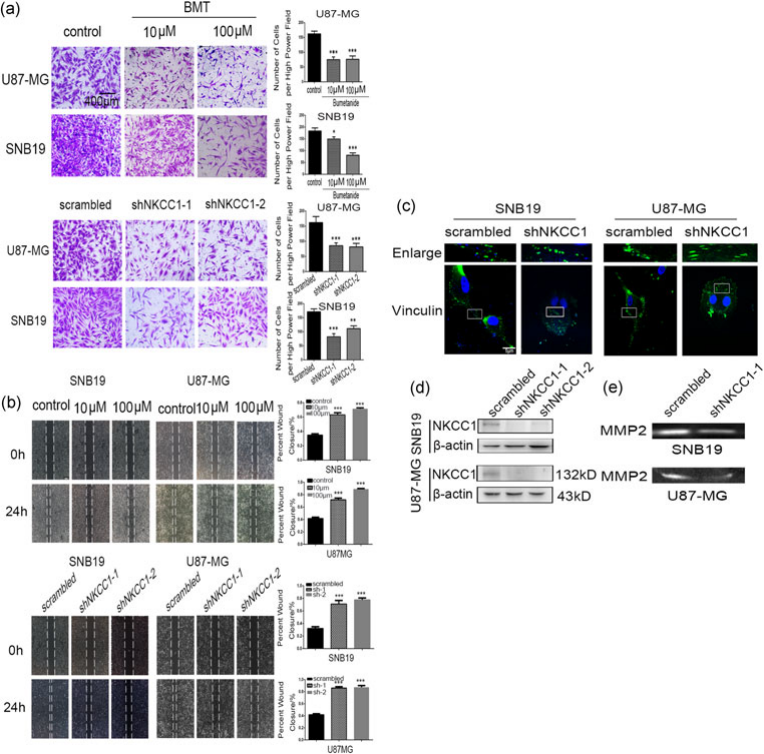
Inhibited NKCC1 decreased the invasion and migration of U87‐MG and SNB19 cells. (a) Glioma cell invasion was assessed with a Transwell assay after the knockdown of NKCC1 or treatment with bumetanide. The scale bar corresponds to 400 μm. (b) A wound healing assay was used to evaluate cell migration ability after knockdown of NKCC1 or treatment with bumetanide after 24 hr. The scale bar corresponds to 500 μm. (c) Immunofluorescent staining for vinculin revealed differences in focal adhesions between the scrambled and NKCC1 knockdown glioma cells. The scale bar corresponds to 5 μm. (d) Silencing of NKCC1 in U87‐MG and SNB19 as detected by western blotting. β‐actin was used as a positive control. (e) MMP2 was detected by western blotting after knockdown of NKCC1. (*p < 0.05; **p < 0.01; ***p < 0.001). MMP2: matrix metallopeptidase 2; NKCC1: sodium‐potassium‐chloride cotransporter 1 [Color figure can be viewed at wileyonlinelibrary.com]

The NKCC1 expression levels in malignant glioma cell lines (NA, U87MG, U251MG, SNB19, LNZ308, LN18, TJ905) were detected by western blotting. The U87‐MG and SNB19 cells exhibited higher levels of expression of NKCC1 than did the NA, U251MG, LNZ308, LN18, TJ905, and LNZ308 cells (Supporting Information Figure S1D). Immunofluorescence staining revealed that NKCC1 was distributed to the leading site of migrating cells (Supporting Information Figure S1A). A CCK‐8 assay (Dojindo Laboratories) was performed to ensure that the drug concentration that we selected in this study did not affect the proliferation abilities of U87‐MG (IC_50_ = 0.6 mM) or SNB19 cells (IC_50_ = 1 mM; Supporting Information Figure S1C). Next, we used BMT, a specific inhibitor of NKCC1 (Lee et al., 2003), to evaluate the invasion and migration abilities of U87‐MG and SNB19 cells as influenced by NKCC1 using a Transwell assay and a wound‐healing assay (Figure 3a,b). Moreover, we used two shRNAs (shNKCC1‐1 and shNKCC1–2) that targeted NKCC1 (Figure 3d). Both shRNA1 and shRNA2 reduced NKCC1 expression in glioma cells compared with the control as assessed by western blotting. To test whether the downregulation of NKCC1 in U87‐MG and SNB19 cells affected their migration and invasion abilities, the wound healing assay and Transwell assay were used and demonstrated that silencing NKCC1 inhibited cell invasion and migration abilities compared with cells that were transfected with scrambled lentiviral vectors (Figure 3a,b). All of the abovementioned data implied that NKCC1 had a critical effect on the migration and invasion of malignant glioma cells.

### 3.4 NKCC1 promoted EMT in U87‐MG and SNB19 cells

**Figure 4:**
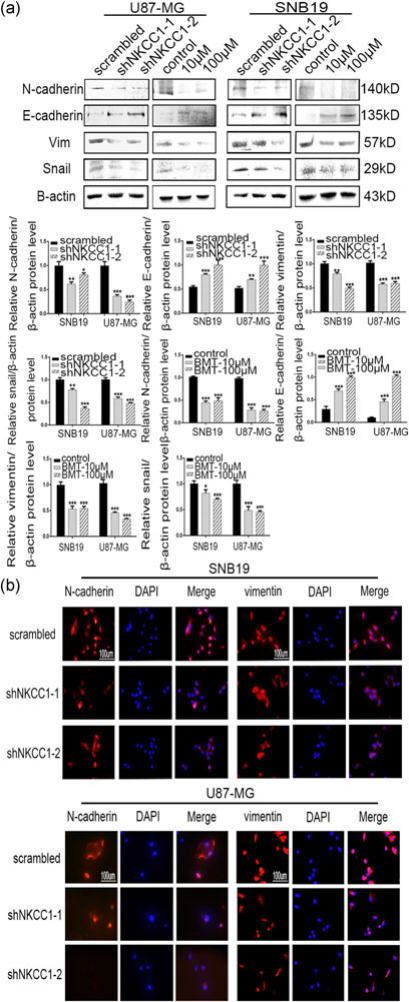
The downregulation of NKCC1 reversed the epithelial–mesenchymal transition. (a) Western blotting showing the decreased protein levels of mesenchymal markers (N‐cadherin, vimentin, and snail) and the increased protein levels of an epithelial marker (E‐cadherin) after knockdown of NKCC1. The same effect was observed after treatment with bumetanide after 24 hr. β‐actin was used as a positive control. (b) Immunofluorescence staining showing the same outcomes as for NKCC1 in U87 and SNB19 cells after shRNA treatment and bumetanide treatment. The scale bar corresponds to 100 μm (*p < 0.05; **p < 0.01; ***p < 0.001). NKCC1: sodium‐potassium‐chloride cotransporter 1 [Color figure can be viewed at wileyonlinelibrary.com]

A single‐cell colon assay was performed to demonstrate that NKCC1 knockdown resulted in greater cell adhesion, the maintenance of more compact contact with neighboring cells, and a tendency toward an epithelial morphology (Supporting Information Figure S2B). Additionally, we demonstrated that the expressions of EMT‐related markers were markedly influenced by BMT or NKCC1 knockdown based on western blot. The results (Figure 4a) revealed that the expression of the epithelial marker E‐cadherin increased with the drug concentration, and the expressions of mesenchymal markers, such N‐cadherin, vimentin and snail, gradually decreased. We also demonstrated that the EMT markers were significantly altered by NKCC1 knockdown (Figure 4a). We further demonstrated the same effect on EMT marker expression with immunofluorescence staining assays (Figure 4b) that demonstrated that the pharmacological inhibition or knockdown of NKCC1 downregulated both N‐cadherin and vimentin in the U87‐MG and SNB19 cells.

EMT is further associated with increased activities of matrix metalloproteases (MMPs), which serve to degrade extracellular matrix proteins and facilitate cell invasion. A gelatin zymography assay was performed to assess the changes in the MMP2 protein level. The results (Figure 3e) revealed significant decreases in the levels of active MMP2 in the U87‐MG and SNB19 cells after the knockdown of NKCC1.

Finally, vinculin immunofluorescent labeling resulted in a punctate or threadlike pattern of small focal adhesions in the control cells (Figure 3c). However, treatment with the shRNA for NKCC1 led to cell rounding and induced large peripheral adhesions, while decreasing the number of adhesions in the center area of the cell (Figure 3c). Taken together, these results suggested that the inhibition of NKCC1 induced morphological, biochemical, and functional changes reminiscent of EMT.

### 3.5 Rac1 and RhoA signaling mediated NKCC1‐induced EMT

**Figure 5:**
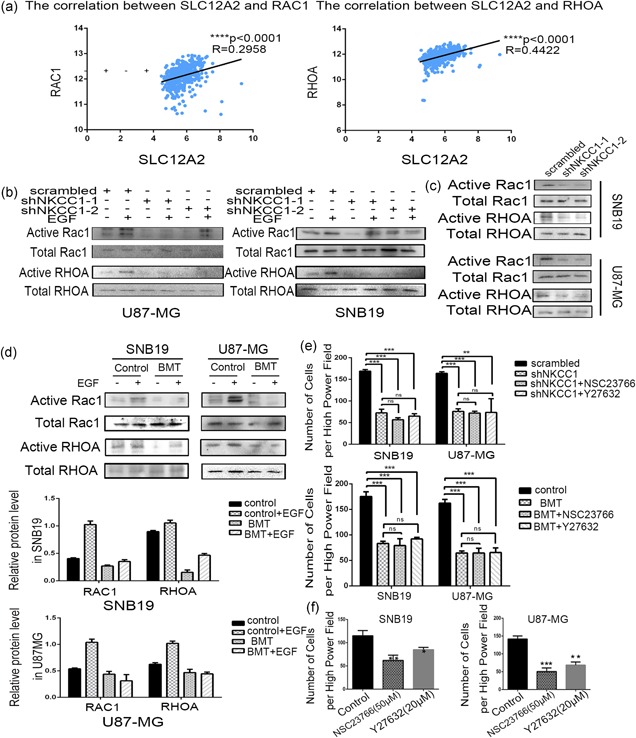
Rac1 and RhoA signaling mediated NKCC1‐induced EMT. (a) Pearson correlation analysis between the SLC12A2 and RAC1/RHOA mRNA expression in TCGA data sets. (b,c) Knockdown NKCC1 decreased actived Rac1/RhoA levels in U87‐MG and SNB19 cells detected by western blotting. (d) Bumetanide (100 μM) treated after 24 hr decreased actived Rac1 and RhoA levels. β‐actin was used as a positive control. (e) A Transwell assay showing the invasion power of U87‐MG and SNB19 cells using NKCC1 knockdown, sh‐ NKCC1 combined with NSC23766 or Y‐27632. A Transwell assay using bumetanide and NSC23766/Y‐27632 combination. (f) NSC23766 and Y‐27632 decreased the invasion of glioma cells detected by a Transwell assay. (*p < 0.05; **p < 0.01; ***p < 0.001). EMT: epithelial–mesenchymal transition; NKCC1: sodium‐potassium‐chloride cotransporter 1; TCGA: The Cancer Genome Atlas [Color figure can be viewed at wileyonlinelibrary.com]

To further investigate the underlying mechanism, the TCGA data set was searched to demonstrate that the expression of the SLC12A2 gene was positively correlated with the expressions of the RAC1 and RHOA genes (Figure 5a; *p* < 0.0001). RhoA and Rac1 belong to a family of GTPases that regulate F‐actin assembly and disassembly while controlling cell migration. As illustrated in Figure 5d, we performed GST‐TRBD and GST‐PBD pull‐down assays, and RhoA and Rac1 activities were decreased basally following BMT treatment in both the U87‐MG and SNB19 cells. EGF stimulation increased the activations of RhoA and Rac1, while treatment with BMT attenuated this activation. Next, we evaluated the activations of RhoA and Rac1 in NKCC1 knockdown U87‐MG and SNB19 cells (Figure 5b,c), and an effect similar to that of BMT was observed. Finally, to implicate these downstream pathways in the regulation of the migration of glioma cells, we treated U87‐MG and SNB19 cells with small molecule inhibitors of Rho‐associated kinase (Y27632) and Rac1 (NSC23766) and assessed the effects on migration using a Transwell migration assay. We found that both compounds significantly reduced the migration of U87‐MG and SNB19 cells (Figure 5f). Furthermore, we simultaneously treated U87‐MG and SNB19 cells with BMT (100 μM) and small molecule inhibitors of Rho‐associated kinase (Y27632) or inhibitors of Rac1 (NSC23766) (Figure 5e). In a similar manner, we applied Y27632 or NSC23766 to NKCC1 knockdown cells or scrambled cells (Figure 5e) to evaluate the migration ability via the Transwell assay, and the results revealed that there was no significant difference between the combined inhibition and the single inhibition of NKCC1. Taken together, these findings suggest that NKCC1 is required for the activation of RhoA and Rac1.

### 3.6 NKCC1 promoted tumor invasion in vivo

**Figure 6:**
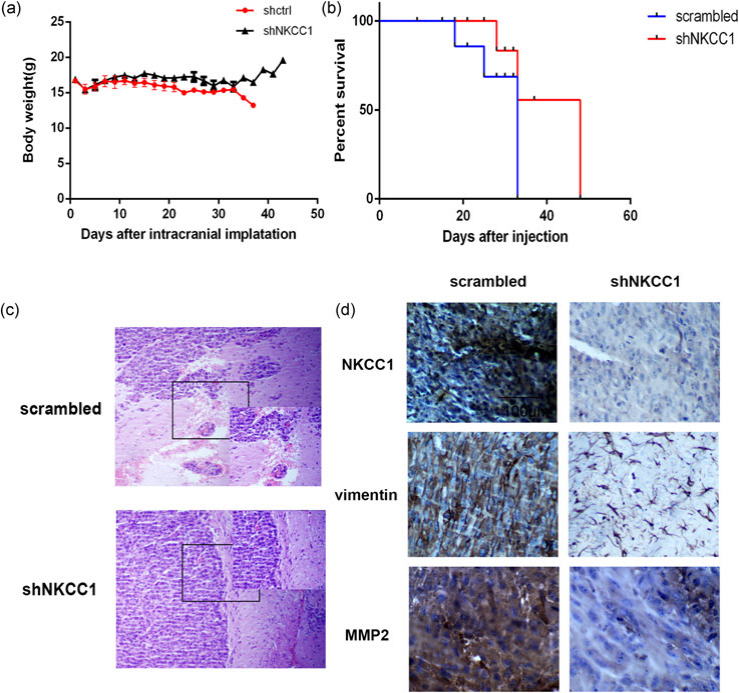
Downregulation of NKCC1 inhibited invasion in intracranial nude mouse. (a) Mouse body weight was recorded as a measure of mouse nutrition in the two groups. (b) A survival curve was used to detect differences in mouse survival times between the two groups. (c,d) Immunohistochemistry analysis of the H&E and expression of NKCC1 and MMP‐2 and vimentin between NKCC1‐shRNA‐treated tumors compared to tumors in the scrambled group. The scale bar corresponds to 100 μm. (*p < 0.05; **p < 0.01; ***p < 0.001). MMP‐2: matrix metallopeptidase 2; NKCC1: sodium‐potassium‐chloride cotransporter 1 [Color figure can be viewed at wileyonlinelibrary.com]

After a preliminary in vitro experiment, we extended our investigation to examine whether NKCC1 knockdown could produce similar effects in vivo. In consideration of the fact that BMT cannot effectively cross the blood brain barrier, we used Lentiviral transfection. After orthotopic implantation of glioma cells into mouse brains, a significant decrease in body weight was observed in the mice of the U87‐scrambled groups (Figure 6a). Additionally, the mice in the U87‐Sh groups exhibited significantly longer survival times (Figure 6b). Furthermore, H&E and IHC analyses revealed that the invasive border of the tumor and the expression of NKCC1, vimentin, and MMP2 were decreased in the U87‐Sh groups (Figure 6c,d), which is consistent with the in vitro results.

## 4 DISCUSSION

GBM is the most aggressive primary brain tumor in adults. However, the mechanisms that confer GBM cells with their invasive behavior are still poorly understood. In this study, we confirmed that the pharmacological inhibition and knockdown of NKCC1 both sharply decreased glioma invasion and migration. Second, we verified that NKCC1 inhibition elicited a series of changes in the expressions of EMT markers, which indicted that NKCC1 promoted the EMT‐like process in gliomas. Third, we also found that NKCC1 regulated EMT through the Rac1 and RhoA signaling pathways in gliomas. NKCC1 inhibition effectively decreased the activation of Rac1 and RhoA, and the pharmacological inhibition of Rac1 and RhoA dramatically impaired glioma invasion and migration. Finally, we demonstrated that the spread of intracranial tumors in NKCC1 knockdown U87‐MG cells was significantly decreased. Therefore, we suggest that the NKCC1 promotes the EMT‐like process in gliomas via the RhoA and Rac1 signaling pathways.

We noticed that GBM patients with surrounding multifocal infiltration and spread exhibited high levels of expression of NKCC1 in the center and border of the tumors. NKCC1 initially received attention as a key tumor cell volume regulator. NKCC1 is widely distributed in various tissues of the human body, is overexpressed in GBM tissue, and is tightly related to many malignancies. Recently, it was reported that high levels of NKCC1 expression predict poor clinical outcomes for lung adenocarcinoma patients and an epidermal growth factor receptor (EGFR)‐mutated subgroup (Sun et al., 2016). In esophageal squamous cell carcinoma, the expression of NKCC1 might be related to the degree of histological differentiation (Shiozaki et al., 2014). A growing amount of data demonstrates that cell migration and invasion are facilitated by ion channels and transporters (Sontheimer, 2008). NKCC1 imbues glioma cells with a great transformation power that enables them to cross the extremely narrow extracellular space; the volume of a migrating glioma cell can be reduced to 30%–50% of the initial volume (Watkins & Sontheimer, 2011). Additionally, in the latest research, NKCC1 has been observed to regulate the cytoskeleton in addition to cell volume. Upon NKCC1 knockdown, there is a decreased expression of Cofilin1 at the plasma membrane coupled with decreases in RhoA and Rac1 activities (Schiapparelli et al., 2017). Therefore, NKCC1 plays a pivotal role in many processes related to the malignant phenotype of gliomas.

We initially verified that NKCC1 promoted the EMT process in gliomas. In our experiment, we found that high NKCC1 expression in glioma cells tended to change their shape and polarity and the cell adhesion with adjacent cells; that is the cell phenotype was changed. Although there are some different features and behavioral patterns between the EMT‐like process in neuroepithelial tumors and the classical EMT process in traditional epithelial tumors, many mesenchymal markers play important roles in glioma malignancy. For example, snail promotes proliferation, migration and invasion through the induction of the EMT process in glioblastomas in vitro (Myung, Choi, Kim, Wang, & Park, 2014). Slug promotes the invasion and migration of gliomas in vitro and facilitates the growth of glioblastomas and angiogenesis in vivo (H.W. Yang, Menon, Black, Carroll, & Johnson, 2010). Moreover, Twist1 accelerates the invasive abilities of GBM by increasing the mesenchymal phenotype (Mikheeva et al., 2010). Therefore, the terms “glial‐to‐mesenchymal transition” or EMT‐like process, which are substitutes for EMT in gliomas, have been increasingly proposed (Iser et al., 2017; Mahabir et al., 2014).

Although GBM is characterized by a locally aggressive pattern, it rarely produces clinically evident extra‐cranial metastases, with only 0.4% of cases experiencing metastases to visceral organs, including the liver, spleen, kidney, and skin (Smith, Hardman, & Earle, 1969). Nevertheless, approximately 20% of GBM patients have detectable levels of circulating tumor cells in their blood (Awan et al., 2015). A recent study demonstrated that single GBM CTCs isolated from both patients and mouse PDX models exhibit enrichment of mesenchymal over neural differentiation markers (Sullivan et al., 2014). Moreover, circulating tumor cells from metastatic breast cancer patients exhibit heterogeneous epithelial and mesenchymal phenotypes, and CTCs display higher levels of the mesenchymal phenotype than carcinoma cells within primary tumors (Yu et al., 2013). Therefore, the mesenchymal transformation is a pivotal molecular event that increases the malignancy of glial tumors (Kahlert, Nikkhah, & Maciaczyk, 2013).

Based on our experimental research, we hypothesized that NKCC1 promotes the EMT‐like process in gliomas via the RhoA and Rac1 signaling pathways. Moreover, numerous studies also support our supposition. Rac1 activation mediates Twist1‐induced cancer cell migration (W.H. Yang et al., 2012). Rac1 overexpression is correlated with the epithelial mesenchymal transition and predicts poor prognosis in non‐small cell lung cancer (Zhou et al., 2016). Rac1 promotes the EMT program in gastric adenocarcinomas and the acquisition of a cancer stem cell state. Rac1 inhibition in gastric adenocarcinoma cells blocks EMT and CSC phenotypes and thus prevents metastasis and may augment chemotherapy (Yoon et al., 2017). These studies and our experimental data provide theoretical support for the mechanism by which NKCC1 promotes EMT.

## 5 CONCLUSIONS

Our research suggests that NKCC1 can regulate glioma cell migration and invasion abilities in vitro and in vivo. We primarily confirmed that NKCC1 promoted the EMT‐like process in gliomas. More important, we confirmed that NKCC1 accelerates EMT in glioblastoma cells via NKCC1‐dependent Rac1/RhoA activation. Our findings also suggest that NKCC1 could serve as a potential new target for the treatment of malignant glioma. Moreover, NKCC1 can be targeted by the FDA‐approved drug BMT, which has been demonstrated to decrease GBM migration in vitro and in vivo (Haas & Sontheimer, 2010). Unfortunately, bumetanide cannot effectively cross the blood brain barrier. Therefore, a new NKCC1 inhibitor that can block NKCC1 in the brain is expected and could be used in combination with the conventional chemotherapy drug temozolomide to disrupt the spread and dissemination of gliomas.

## CONFLICTS OF INTEREST

The authors declare that they have no conflicts of interest.

## AUTHOR CONTRIBUTIONS

H. W. M. performed the experiments and drafted the manuscript. T. L. and Z. N. T. participated in the design of this study. L. H., L. Q. T. and L. Y. participated in the experiments. P. D. L., Y. X., J. B. L., F. Y., C. Z. and H. L. M. contributed to the design of this study, I. R. A. and Y. H. Y. contributed to the final data analysis and edited the manuscript. X. J. Y. and S. P. Y. managed the experimental design, reviewed the manuscript and gave funding support. All authors read and approved the final manuscript.

## Supporting information

Supporting information

Supporting information
