# Supplementary figures and images for: NKCC1 promotes EMT‐like process in GBM via RhoA and Rac1 signaling pathways
Source: J Cell Physiol. 2018 Aug 29;234(2):1630–42. doi: 10.1002/jcp.27033 (PMC6282979; doi:10.1002/jcp.27033)

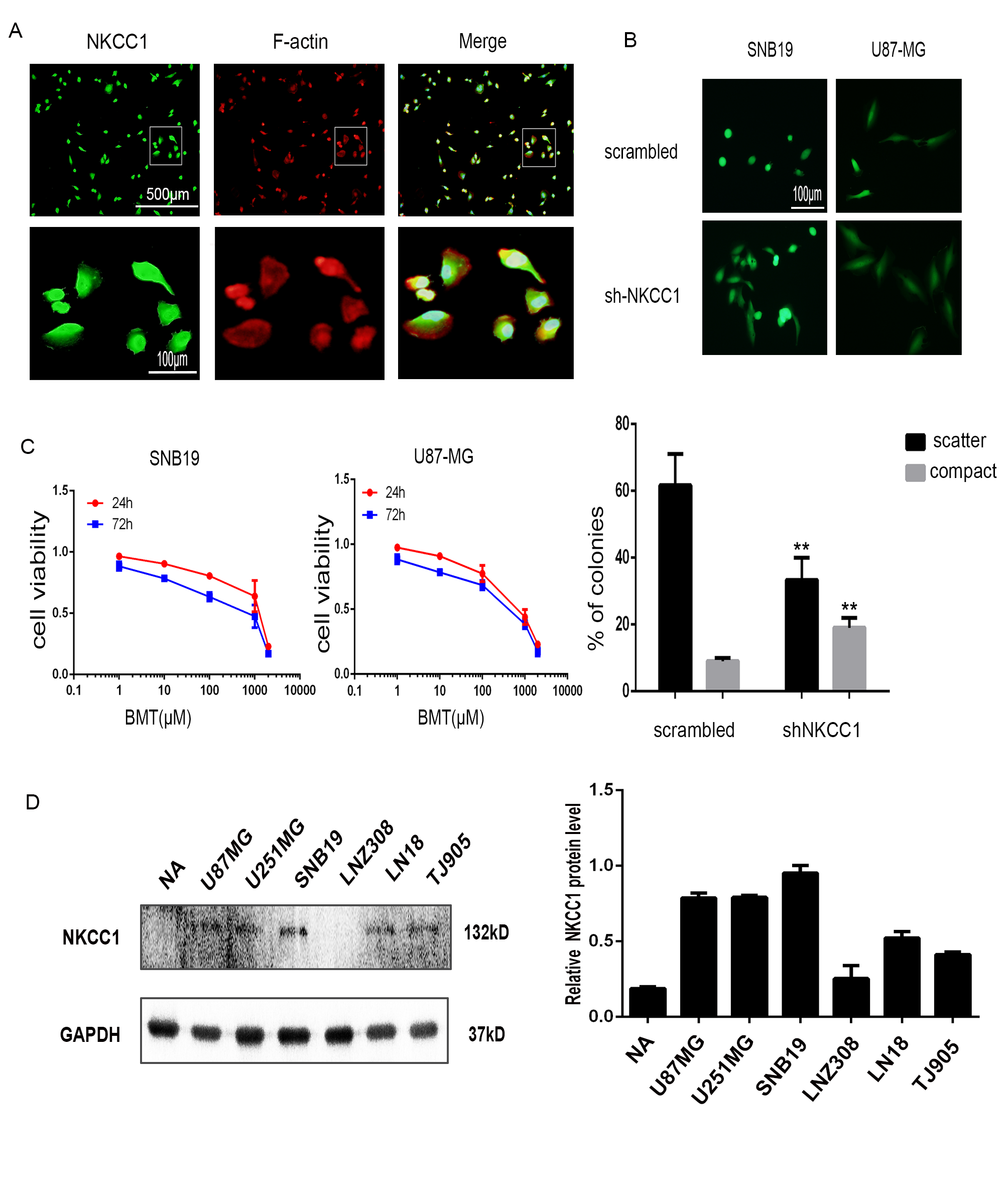

Supplement: Supplementary file 1 — Supporting information [file JCP-234-1630-s001.tif]

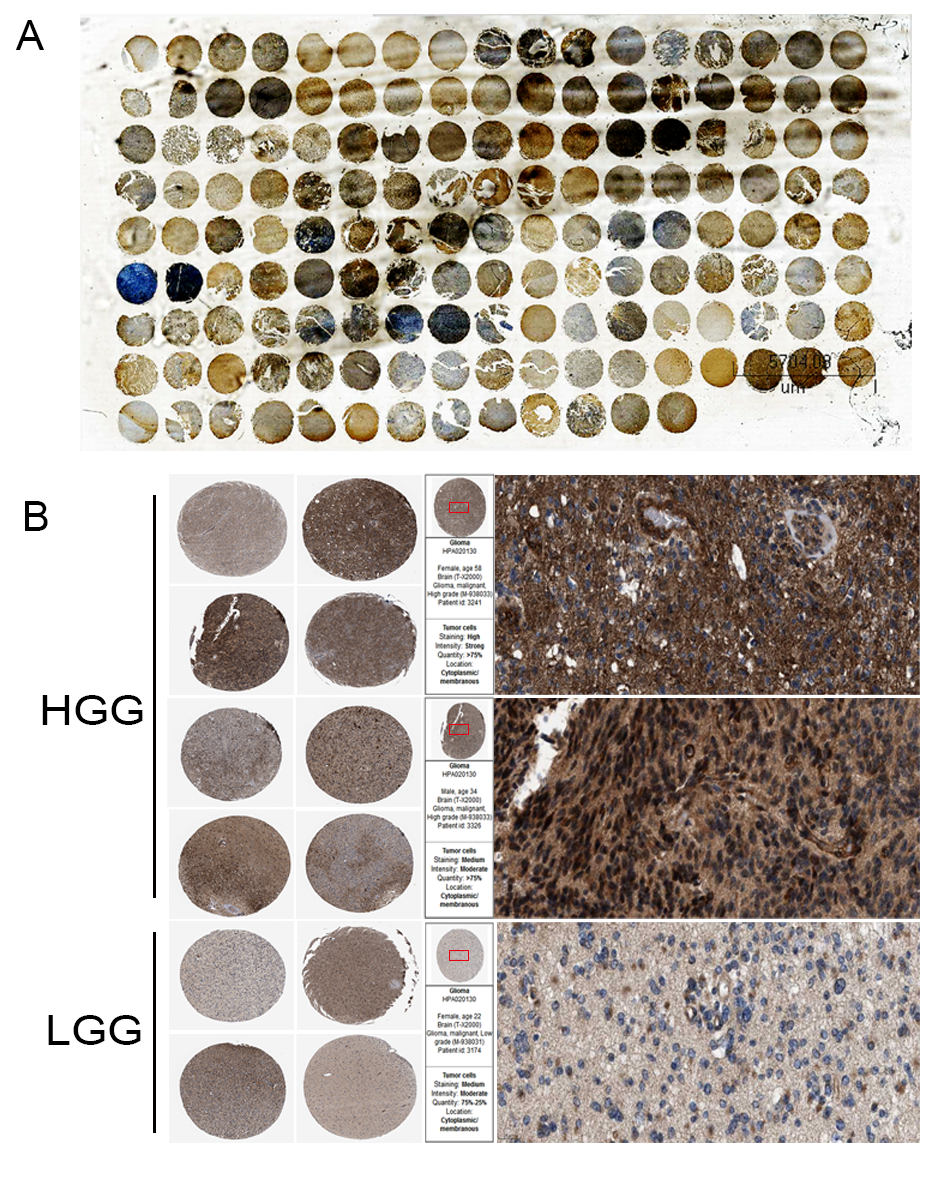

Supplement: Supplementary file 2 — Supporting information [file JCP-234-1630-s002.tif]
